# *Rhus verniciflua* stokes extract, a traditional herbal medicine, combined with first-line chemotherapy for unresectable locally advanced and metastatic pancreatic cancer: a prospective observational pilot study

**DOI:** 10.3389/fonc.2024.1469616

**Published:** 2024-11-14

**Authors:** Hayun Jin, Su Hyeon Lee, Eun Hye Kim, Su Bin Park, Namyoung Park, Kwang Ro Joo, Seong Woo Yoon

**Affiliations:** ^1^ Department of Korean Internal Medicine, Kyung Hee University College of Korean Medicine, Kyung Hee University Hospital at Gangdong, Seoul, Republic of Korea; ^2^ Department of Gastroenterology, Kyung Hee University College of Medicine, Kyung Hee University Hospital at Gangdong, Seoul, Republic of Korea

**Keywords:** *Rhus verniciflua* Stokes, traditional herbal medicine, pancreatic cancer, chemotherapy, advanced cancer

## Abstract

Pancreatic cancer is among the most lethal cancers with limited treatment options. Traditional herbal medicines have been explored as potential adjunct therapies for enhancing the effectiveness of standard chemotherapies. In this study, we investigated the safety and efficacy of *Rhus verniciflua* Stokes (RVS) in combination with first-line chemotherapy for unresectable locally advanced and metastatic pancreatic cancer in a prospective setting. This prospective observational pilot study enrolled patients with confirmed inoperable stage III or IV pancreatic cancer undergoing or scheduled to receive 5-fluorouracil-based or gemcitabine-based first-line chemotherapy with RVS treatment and were followed up for up to 20 months. The primary endpoint was the safety profile of RVS, which was assessed through adverse events. The secondary endpoints included overall response rate (ORR), disease control rate (DCR), progression-free survival (PFS), and overall survival (OS). Cox regression analysis identified possible prognostic factors for PFS and OS. Of the enrolled 25 patients, 18 completed the follow-up. The median RVS treatment duration was 6.14 months (range 2.23–8.01 months) and the median RVS daily dose was 3.8 capsules/day (range 2.1–5.7 capsules/day). RVS showed a favorable safety profile, with only one case of non-severe pruritus possibly related to RVS treatment. No hepatotoxicity and nephrotoxicity related to RVS was reported. The ORR and DCR were 5.6% and 72.2%, respectively. The median PFS and OS were 7.24 months (95% CI: 3.15–12.9) and 13.9 months (95% CI: 1.14–27.72), respectively. Cox regression analysis showed that baseline CA19-9 level was an independent prognostic factor for OS, and the daily dose of RVS was an independent prognostic factor for PFS and OS. In conclusion, RVS can be administered safely to patients with unresectable locally advanced metastatic pancreatic cancer who are undergoing first-line chemotherapy, and the daily dose was an independent prognostic factor for cancer survival. Further randomized controlled studies are warranted to confirm the exact benefits of RVS treatment.

**Clinical trial registration:**

https://cris.nih.go.kr, identifier KCT0007496.

## 1 Introduction

Pancreatic cancer is one of the most fatal human cancers, with localized and advanced cases having a 5-year survival rate of 32% and 12%, respectively (1). Surgical resection, when possible, offers the best chance for cure; however, most patients are typically diagnosed at advanced or metastatic stages when surgery is not feasible, leaving systemic chemotherapy as the only treatment option. Recommended cytotoxic chemotherapy regimens, including gemcitabine- or fluoropyrimidine-based regimens, provide modest clinical benefits owing to the aggressive nature of the disease (2). Additionally, chemotherapy toxicity and resulting decline in the quality of life further limit treatment options throughout the disease course.

Herbal medicines have long been studied as potential sources of supplementary anticancer treatments and are commonly used in patients with cancer worldwide (3). Modern approaches to traditional East Asian medicines have found that they are not only effective in alleviating cancer-related and cancer therapy-related symptoms but are also potentially synergistic when combined with modern anticancer therapeutics (4, 5). However, safety concerns arising from possible herb–drug interactions and herb-induced liver injury must be addressed as patients commonly take herbal medications with chemotherapy (6). To ensure the safe use of herbal medicines and overcome any risks arising from unsupervised medical herbal use, efforts are being made to incorporate traditional herbal medicine treatment into conventional clinical oncology settings (7).

*Rhus verniciflua* Stokes (RVS) has long been used in traditional East Asian medicine to treat tumors arising from organs in the abdominopelvic cavity (8). Prior to the development of modern herbal pharmacological processing, its practical use was limited because of the strong allergenic properties of urushiol, one of the main compounds. Owing to the development of methods to filter strongly allergenic compounds, RVS extract can now be safely administered to patients. Previous retrospective studies and case reports have suggested anticancer potential in various cancer types, including pancreatic cancer (9–12).

Our research team previously conducted a single-center retrospective study on patients with metastatic pancreatic cancer and found that RVS combined with chemotherapy may be effective in improving survival outcomes, although a few non-severe, reversible, and self-limiting adverse events were reported (13). Nonetheless, thorough examination in a prospective setting is required to verify the safety and efficacy of RVS.

In this prospective observational pilot study, we aimed to evaluate the safety and efficacy of RVS extract in patients with unresectable, locally advanced, metastatic pancreatic cancer undergoing standard first-line chemotherapy.

## 2 Materials and methods

### 2.1 Study design

This clinical trial was designed as a prospective, observational, single-arm, pilot study. Patients were recruited from a single hospital located in Seoul, Republic of Korea. The target sample size was 30 participants considering the feasibility of recruitment and available resources. The schedule of visits was determined according to the chemotherapy schedule. Specifically, each visit was conducted at the beginning of each chemotherapy cycle. The patients were followed up for a maximum duration of 20 months.

### 2.2 Patient eligibility

Patients were eligible if they were 19 years or older, had a pathologically confirmed diagnosis of pancreatic cancer, had an inoperable disease status according to the National Comprehensive Cancer Network Clinical Practice Guidelines in Oncology version 1.2019, were scheduled to start or had started within 2 months of first-line 5-fluorouracil (5-FU) or gemcitabine-based chemotherapy, had an Eastern Cooperative Oncology Group (ECOG) performance score of 0 to 2, and had a life expectancy of more than 3 months. Patients were excluded if they were pregnant or breastfeeding, had brain metastasis with clinically significant neurological symptoms or signs, had clinically significant skeletal-related events requiring radiation therapy or surgery, or had been diagnosed with another primary cancer within the past 5 years.

5-FU-based chemotherapy regimens included 5-FU plus leucovorin, FOLFOX, and FOLFIRINOX regimens. Gemcitabine-based chemotherapy regimens included gemcitabine monotherapy, gemcitabine plus nanoparticle albumin-bound paclitaxel (GNP), gemcitabine plus cisplatin, and gemcitabine plus capecitabine. Regimens could be adjusted based on findings of disease progression after response evaluation. Salvage therapies and other treatments were also used during the study if required.

This study was approved by the Institutional Review Board of Kyung Hee University Hospital at Gangdong, Seoul, Republic of Korea (KHNMCOH 2020-11-012). The study was conducted in accordance with Good Clinical Practice guidelines and the principles of the Declaration of Helsinki. All patients provided written informed consent.

### 2.3 Traditional herbal medicine

RVS in the form of a capsuled extract, manufactured at Kyung Hee University Medical Center, was administered to all enrolled patients concurrently with chemotherapy. RVS was roasted for an hour at a 180°C, extracted using distilled water for 2 h, and filtered to remove allergens. The extract was concentrated under vacuum and lyophilized to obtain a powder. Each capsule contained 450 mg of the powdered extract. The quality of the RVS extract was tested and monitored according to the standards of the investigating site (fisetin>0.6%; urushiol not detected).

RVS was prescribed by licensed and experienced traditional Korean medicine doctors at Kyung Hee University Medical Center at Gangdong. Included patients received RVS extract at the typical dose of 1 or 2 capsules per administration, 2 or 3 times daily, and 30 min after a meal, according to patient compliance and preference. Doses were recorded at each visit. The administration of salvage therapies using Korean medicines was allowed, if necessary, to manage cancer treatment-related symptoms based on findings from previous studies (13, 14).

### 2.4 Outcome measurement

The primary endpoint of this study was safety of RVS. The occurrence of any adverse events during the study period was recorded at each visit using the Common Terminology Criteria for Adverse Events version 5.0, and causality was assessed using the WHO-UMC causality assessment system. The liver and renal profiles were measured and compared before the initiation of RVS treatment and after the completion of RVS treatment.

The secondary endpoints were tumor response and survival, including the overall response rate (ORR), disease control rate (DCR), progression-free survival (PFS), overall survival (OS), and disease-related OS. ORR was defined as the percentage of patients who achieved complete response (CR) and partial response (PR) during the treatment course, whereas DCR was defined as the percentage of patients who achieved CR, PR, and stable disease (SD) (15). All tumor responses were evaluated according to the Response Evaluation Criteria in Solid Tumors (RECIST) version 1.1.

Survival outcomes, including unreported deaths or disease progression, were documented by monitoring the electronic medical records of our hospital or by contacting the patients by telephone every 4 weeks. PFS was defined as the time from the initiation of chemotherapy to disease progression or death from any cause. OS was defined as the time from the initiation of chemotherapy to death from any cause.

### 2.5 Statistical analysis

The outcomes of patients who completed the RVS treatment were analyzed. Adverse events are reported using descriptive statistics in the form of frequencies and percentages. For survival data, Kaplan–Meier estimates were used to calculate the median survival time and plot the data. Univariate Cox regression analyses for sex, age, tumor stage, number of metastases, ECOG performance score, baseline serum CA 19-9 level, Charlson comorbidity index, presence of > 20% chemotherapy dose reduction, and RVS dose were performed to search for possible prognostic factors for PFS and OS. Variables that showed statistical significance of *p*<0.2 were considered potentially significant and were used to perform multivariate Cox analysis. Paired *t*-tests were performed to test for differences in continuous variables before and after chemotherapy. All tests were two-sided, and *p*<0.05 was considered statistically significant. All statistical analyses were performed using R: A language and environment for statistical computing version 4.3.3 (R Foundation, Vienna, Austria).

## 3 Results

### 3.1 Demographic and clinical characteristics

**Figure 1:**
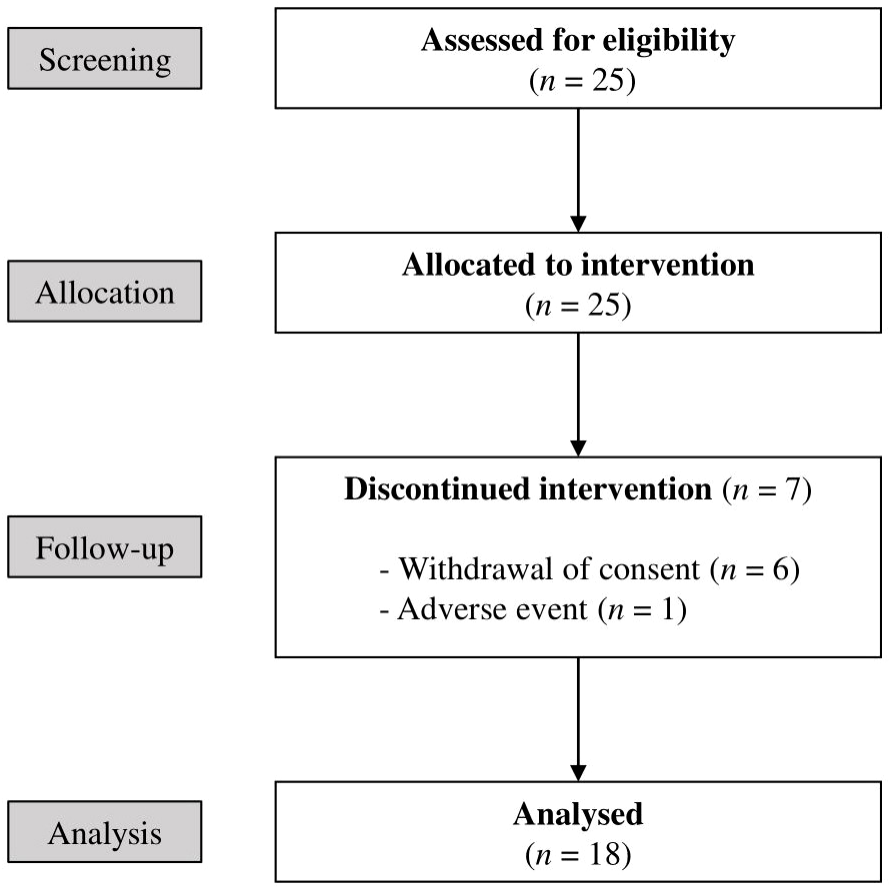
CONSORT flow diagram of the study.

Between December 2020 and December 2022, 25 patients who met the eligibility criteria were enrolled in this study and received chemotherapy in combination with RVS treatment. Throughout the study, 7 patients dropped out, and 18 patients completed the follow-up. Reasons for dropping out included withdrawal of consent in six cases and the occurrence of adverse event in one case. The CONSORT diagram for the flow of the study is depicted in **Figure 1**.

The baseline patient characteristics are listed in **Table 1**. The mean age of the patients was 65 ± 9.68 years. Nine (50%) of the eighteen patients were male. Twelve patients (66.7%) had stage IV, and six (33.3%) had stage III disease. The tumors were located in the head of the pancreas in 11 patients (61.1%), and the median tumor size was 4.8 cm (range 2.8–7.6 cm). Sixteen patients (88.9%) had an ECOG performance status of 0 to 1. For initial chemotherapy, 16 (88.9%) patients received FOLFIRINOX, and 2 (11.1%) underwent GNP regimens. The median follow-up duration was 11.75 months. The median number of first-line chemotherapy cycles was 9 for FOLFIRINOX and 4.5 for GNP. Of the 18 patients, 8 (44.4%) received second-line chemotherapy.

**Table 1:** Baseline characteristics of the patients.

Characteristics	n (%)
Age, years, median (range)	65 (51–81)
<65	8 (44.4)
≥65	10 (55.6)
Sex	
Male	9 (50)
Female	9 (50)
Comorbidities	
Diabetes mellitus	13 (72.2)
Hypertension	10 (55.6)
Liver disease	2 (11.1)
Tumor stage	
Stage III	6 (33.3)
Stage IV	12 (66.7)
Tumor location	
Head	11 (61.1)
Tail	5 (27.8)
Body	2 (11.1)
Tumor size, median (range)	
0–3 cm	1 (8.3)
3–6 cm	9 (75)
6 cm~	2 (16.7)
Sites of metastasis	
Liver	7 (58.3)
Lung	4 (33.3)
Distant lymph nodes	3 (25)
Common bile duct	2 (16.7)
Peritoneum	2 (16.7)
Bone	1 (8.3)
Initial chemotherapy regimen	
FOLFIRINOX	16 (88.9)
Gemcitabine plus nab-paclitaxel	2 (11.1)
ECOG performance score	
0–1	16 (88.9)
2	2 (11.1)

ECOG, Eastern Cooperative Oncology Group.

RVS was administered for a median of 6.14 months (range 2.23–8.01 months). The median average daily dose was 3.8 capsules/day (range 2.1–5.7 capsules/day) or 1721.5 mg/day (range 936.6–2581.6 mg/day). Two patients received astragalus-based salvage Korean medicine to control cancer-related anorexia and fatigue.

### 3.2 Safety

One patient dropped out after less than a month of RVS treatment after reporting an adverse event possibly related to the treatment. The patient reported grade II pruritus on the day of the initial oral administration of RVS. Three days after onset, the symptoms resolved spontaneously without complications. Otherwise, no adverse events related to RVS treatment were observed in the 18 patients analyzed.

The incidence rates of adverse events during the study period are shown in **Table 2**. Grade III and IV adverse events were reported in 10 patients. The hematologic adverse events included eight cases of neutropenia and one case of anemia. Non-hematologic grade III and IV adverse events included ascites, bile duct stenosis, oral mucositis, infection, hypoxia, and spinal fracture.

**Table 2:** Adverse events by types and grades.

Incidence	Grade I, II	Grade III	Grade IV
Hematologic			
Neutropenia	1 (5.6%)	6 (33.3%)	3 (16.7%)
Anemia	0 (0%)	1 (5.6%)	0 (0%)
Non-hematologic			
Generalized weakness	5 (27.8%)	0 (0%)	0 (0%)
Vomiting	2 (11.1%)	0% (0)	0 (0%)
Nausea	1 (5.6%)	0 (0%)	0 (0%)
Ascites	0 (0%)	2 (11.1%)	0 (0%)
Oral mucositis	0 (0%)	1 (5.6%)	0 (0%)
Bile duct stenosis	0 (0%)	0 (0%)	1 (5.6%)
Infection	0 (0%)	1 (5.6%)	0 (0%)
Fever	1 (5.6%)	0 (0%)	0 (0%)
Aspiration	0 (0%)	1 (5.6%)	0 (0%)
Hypoxia	0 (0%)	1 (5.6%)	0 (0%)
Thromboembolic event	1 (5.6%)	0 (0%)	0 (0%)
Hypokalemia	1 (5.6%)	0 (0%)	0 (0%)
Spinal fracture	0 (0%)	1 (5.6%)	0 (0%)
Dysuria	1 (5.6%)	0 (0%)	0 (0%)
Injection site reaction	1 (5.6%)	0 (0%)	0 (0%)

To further evaluate the possibility of drug-induced hepatotoxicity and nephrotoxicity of RVS, we compared seven laboratory biomarkers before and after RVS treatment: total bilirubin, aspartate aminotransferase, alanine aminotransferase, alkaline phosphatase, gamma-glutamyl peptidase, blood urea nitrogen, and creatinine. Statistical analysis of these markers revealed no significant differences before and after RVS treatment, implying that RVS is unlikely to cause hepatotoxicity or nephrotoxicity. The results are summarized in **Table 3**.

**Table 3:** Changes in liver and renal function test.

	Before RVS treatment	After RVS treatment	*P*-value
Total bilirubin	0.95 ± 0.85	0.87 ± 1.08	0.78
AST	35.81 ± 28.97	37.81 ± 30.73	0.68
ALT	36 ± 31.41	32.19 ± 28.53	0.71
ALP	192.07 ± 246.19	240.2 ± 241.80	0.44
GGT	156.57 ± 252.88	184.43 ± 280.3	0.53
BUN	13.33 ± 5.15	16.53 ± 9.50	0.08
Cr	0.71 ± 0.19	0.72 ± 0.16	0.30

RVS, *Rhus verniciflua* stokes; AST, aspartate aminotransferase; ALT, alanine aminotransferase; ALP, alkaline phosphatase; GGT, gamma-glutamyl peptidase; BUN, blood urea nitrogen; Cr, creatinine.

### 3.3 Tumor response and survival

ORR and DCR were assessed according to RECIST version 1.1. No patients achieved CR, 1 (5.6%) patient achieved PR, 12 (66.7%) patients had SD, and 3 (16.5%) patients had progressive disease. The ORR and DCR were 5.6% and 72.2%, respectively.

**Figure 2:**
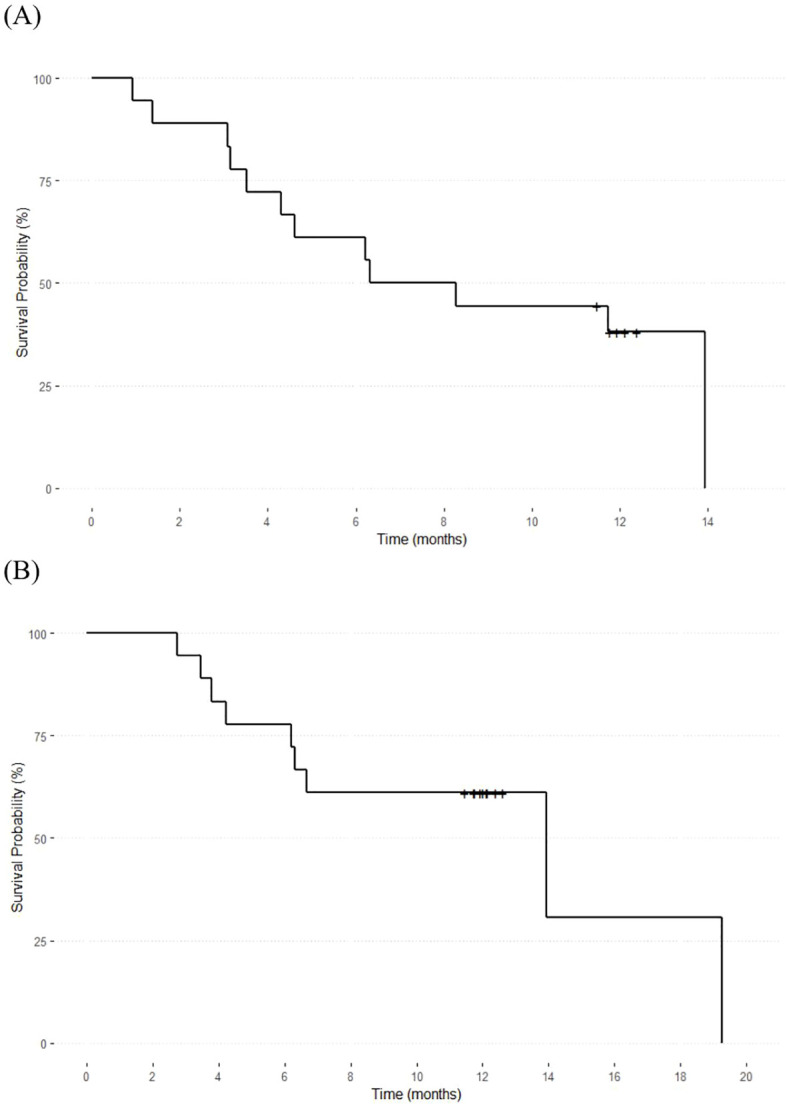
Overall survival and progression-free survival of the included patients. The median overall survival **(A)** was 13.9 months (95% CI: 1.14–27.72), and the median progression-free survival **(B)** was 7.24 months (95% CI: 3.15–12.9).

The Kaplan–Meier survival curves depicting the PFS and OS of the included patients are shown in **Figure 2**. The median PFS was 7.24 months (95% confidence interval [CI]: 3.15–12.9) and the median OS was 13.9 months (95% CI: 1.14–27.72). All deaths were caused by disease progression. Therefore, disease-related OS was equal to OS.

### 3.4 Cox regression analysis

Results of univariate and multivariate Cox regression analysis are presented in **Table 4**. Univariate analysis of PFS showed that the number of metastases, baseline serum CA19-9 level, and daily RVS dose might be predictors of survival (*p*<0.2); however, only daily RVS dose showed statistical significance (hazard ratio [HR] 0.18, 95% CI: 0.03–0.94, *p*=0.04) as an independent prognostic factor for PFS in the multivariate analysis.

**Table 4:** Univariate and multivariate analysis of factors associated with overall survival and progression-free survival.

Variable	*n*	Univariate analysis				Multivariate analysis			
		PFS		OS		PFS		OS	
		HR (95% CI)	*P*-value	HR (95% CI)	*P*-value	HR (95% CI)	*P*-value	HR (95% CI)	*P*-value
Sex									
Female	9	1							
Male	9	0.53 (0.16–1.74)	0.30	1.26 (0.33–4.73)	0.73				
Age group (years)									
<65	8	1							
≥65	10	1.61 (0.51–5.11)	0.42	0.50 (0.12–2.03)	0.34				
Tumor stage									
Stage III	6	1							
Stage IV	12	1.93 (0.52–7.07)	0.32	1.20 (0.23–6.20)	0.83				
No. of distant metastases	12	1.49 (0.88–2.54)	0.14	1.41 (0.73–2.73)	0.31	1.91 (0.93–3.95)	0.08		
ECOG	18	2.78 (0.57–13.50)	0.20	3.78 (0.69–20.81)	0.13			0.75 (0.02–24.59)	0.87
CA 19–9									
<1000 U/mL	11	1		1		1		1	
≥1000 U/mL	5	2.62 (0.71–9.63)	0.15	6.98 (1.26–38.63)	0.03*	3.11 (0.72–13.43)	0.13	35.52 (2.26–557.69)	0.01*
CCI	18	1.10 (0.87–1.38)	0.42	0.96 (0.72–1.29)	0.78				
DR >20%									
No	8	1		1					
Yes	10	1.23 (0.40–3.79)	0.72	0.48 (0.11–2.13)	0.33				
RVS daily dose	18	0.30 (0.08–1.12)	0.07	0.09 (0.01–0.57)	0.01*	0.18 (0.03–0.94)	0.04*	0.01 (0.00–0.54)	0.02*

^*^ *p*<0.05.

ECOG, Eastern Cooperative Oncology Group; CCI, Charlson Comorbidity Index; DR, chemotherapy dose reduction; PFS, progression-free survival; OS, overall survival; HR, hazard ratio; RVS, *Rhus verniciflua* stokes.

Univariate analysis of OS showed that ECOG performance status, baseline serum CA 19-9 level, and daily RVS dose might be predictors of survival (*p*<0.2). Multivariate analysis of the three variables showed that baseline serum CA19-9 level≥1000 was associated with significantly poorer survival (HR 35.52, 95% CI: 2.26–557.69, *p*=0.01) while a higher average daily dose of RVS was associated with significantly better survival (HR 0.01, 95% CI: 0.00–0.54, *p*=0.02) as independent prognostic factors for OS.

## 4 Discussion

Our results showed that the RVS extract can be safely and effectively administered to patients with advanced and metastatic pancreatic cancer undergoing first-line gemcitabine- or fluoropyrimidine-based chemotherapy. To the best of our knowledge, this is the first prospective clinical study of RVS in patients with cancer.

In this study, administration of RVS throughout and after chemotherapy appeared to be generally safe. However, the single adverse event of pruritus, possibly related to RVS, implies that careful monitoring after the initiation of RVS therapy is needed. Non-severe pruritus of the skin has rarely been reported in previous studies (9, 10). Although all the RVS compounds were processed to remove urushiol, the patient may have been hypersensitive to other compounds in the RVS extract.

Furthermore, the toxicity profiles of combined chemotherapy and RVS treatment were mostly consistent with the previously reported profiles of FOLFIRINOX and GNP, with notably lower rates of vomiting (16). Moreover, RVS extract did not induce hepatotoxicity or nephrotoxicity. Therefore, we concluded that RVS neither had significant toxicity of its own nor increased the effects of chemotherapeutic toxicity.

Recent clinical studies concerning survival outcomes of advanced and metastatic pancreatic cancer reported median PFS ranging from 3.3 to 9.4 months, and median OS ranging from 6.8 to 13.9 months depending on the chemotherapy regimens (16–19). A recent study based on the Korean Pancreatic Cancer Registry analyzed data from 413 metastatic pancreatic cancer patients and showed PFS of 7.5 months (FOLFIRINOX) *vs* 8.1 months (GNP) and OS of 11.5 months (FOLFIRINOX) *vs* 12.7 months (GNP) (16). Although head-to-head comparisons of outcomes with this study are not feasible, the results from our study with a PFS of 6.31 months and OS of 13.93 months showed consistency in survival outcomes compared with that of previous studies.

Herb-drug interaction of RVS with chemotherapeutic drugs is an important clinical aspect that should be considered. Herb-drug interaction usually presents in form of altered pharmacokinetics of administered drugs leading to increased toxicity and/or decreased efficacy of treatment. Recent preclinical studies have suggested that RVS may affect the activity of certain cytochrome P (CYP) enzymes that play key roles in human drug metabolism in the liver. Specifically, RVS was shown to have inhibitory effects on CYP2C9, CYP2C19, and CYP1A2 (20). Oxaliplatin, which is used in various chemotherapy regimens, including FOLFIRINOX, for the treatment of pancreatic cancer, has also been shown to have an inhibitory effect on CYP2C9 (21). Although no significant adverse events have been observed in previous clinical settings and in our study, since co-inhibition of a certain CYP enzyme may lead to pharmacokinetic drug-drug interactions, further pharmacokinetic studies are needed to verify the safety of the co-administration of RVS and chemotherapeutics.

In this study, a higher average daily dose of RVS was associated with significantly better survival outcomes. In our previous single-center retrospective study of metastatic pancreatic cancer patients, RVS-based traditional herbal medicine treatment combined with chemotherapy for more than 30 days resulted in significantly longer OS than that of chemotherapy alone, and this combined treatment for more than 30 days was a significant independent prognostic factor for OS (13). Further prospective randomized controlled trials with head-to-head comparisons of the efficacy and safety of different RVS doses are needed.

Compelling clinical evidence has demonstrated that herbal medicines play a positive role in pancreatic cancer when administered along with conventional chemotherapy. A case series and a cohort study reported that combined treatment of Chinese herbal medicine and conventional treatment may lead to a better overall prognosis than conventional treatment alone in patients with pancreatic cancer (22, 23). A phase II clinical trial revealed that a phytosome complex product of the herbal compound curcumin may be effective in improving the survival outcomes of pancreatic cancer patients undergoing gemcitabine monotherapy and demonstrated that concurrent administration of curcumin may be as beneficial as adding another chemotherapeutic agent without significantly increasing the toxicity profile in patients (24).

The anticancer effects of RVS have been reported to be related to the Janus kinase/signal transducer and activator of transcription (JAK/STAT) pathway in pancreatic cancer cells (25). The JAK/STAT pathway, which plays a role in cellular proliferation, organ development, and immune homeostasis, has recently been highlighted as a key pathway in human malignancies including pancreatic cancer. By targeting the JAK/STAT pathway, RVS may be effective in inhibiting cancer cell proliferation and metastasis and increasing chemosensitivity of cancer cells (26, 27). This might result in better clinical outcomes not only for patients receiving chemotherapeutic treatment but also for those who cannot undergo chemotherapy due to poor performance or drug intolerability.

This study has some limitations. First, because this was a single-arm pilot study with a small sample size and limited diversity, the results cannot be applied directly to the general population. Second, the efficacy of RVS combined with chemotherapy could not be directly compared to conventional chemotherapy alone. Third, because this was an observational study, the RVS dose was determined based on typical clinical practice by experienced traditional Korean medicine doctors, and the optimum RVS dose could not be established. Finally, because the RVS dose was decided based on patient compliance and preference, it may have been affected by the general performance of the patients. Since patient performance is an independent prognostic factor for survival, the relationship between the RVS dose and survival outcomes suggested in this study should be carefully interpreted as confounding factors that might have caused bias.

In conclusion, RVS combined with chemotherapy can be safely administered to patients with advanced or metastatic pancreatic cancer and may be beneficial for prolonging survival. Larger randomized clinical trials with robust designs are required to confirm the efficacy, and pharmacokinetic studies are also needed to establish the safety.

## Data Availability

The original contributions presented in the study are included in the article/supplementary material. Further inquiries can be directed to the corresponding authors.
